# Governance for anti-racist AI in healthcare: integrating racism-related stress in psychiatric algorithms for Black Americans

**DOI:** 10.3389/fdgth.2025.1492736

**Published:** 2025-05-15

**Authors:** Christopher T. Fields, Carmen Black, Jannat K. Thind, Oluwole Jegede, Damla Aksen, Matthew Rosenblatt, Shervin Assari, Chyrell Bellamy, Elijah Anderson, Avram Holmes, Dustin Scheinost

**Affiliations:** ^1^ Program for Recovery and Community Health, Department of Psychiatry, Yale School of Medicine, New Haven, CT, United States; ^2^ Magnetic Resonance Research Center, Department of Radiology and Biomedical Imaging, Yale School of Medicine, New Haven, CT, United States; ^3^ Institute of Living at Hartford Hospital, Hartford, CT, United States; ^4^ Department of Psychiatry, Yale School of Medicine, New Haven, CT, United States; ^5^ Equity Research and Innovation Center (ERIC), Yale School of Medicine, New Haven, CT, United States; ^6^ Department of Family Medicine, Charles R Drew University of Medicine and Science, Los Angeles, CA, United States; ^7^ Department of Sociology, Yale University, New Haven, CT, United States; ^8^ Department of Psychiatry, Brain Health Institute, Rutgers University, Piscataway, NJ, United States

**Keywords:** anti-racist AI, racism-related stress, clinical algorithms, algorithmic bias, community-based participatory research

## Abstract

While the world is aware of America's history of enslavement, the ongoing impact of anti-Black racism in the United States remains underemphasized in health intervention modeling. This Perspective argues that algorithmic bias—manifested in the worsened performance of clinical algorithms for Black vs. white patients—is significantly driven by the failure to model the cumulative impacts of racism-related stress, particularly racial heteroscedasticity. Racial heteroscedasticity refers to the unequal variance in health outcomes and algorithmic predictions across racial groups, driven by differential exposure to racism-related stress. This may be particularly salient for Black Americans, where anti-Black bias has wide-ranging impacts that interact with differing backgrounds of generational trauma, socioeconomic status, and other social factors, promoting unaccounted for sources of variance that are not easily captured with a blanket “race” factor. Not accounting for these factors deteriorates performance for these clinical algorithms for all Black patients. We outline key principles for anti-racist AI governance in healthcare, including: (1) mandating the inclusion of Black researchers and community members in AI development; (2) implementing rigorous audits to assess anti-Black bias; (3) requiring transparency in how algorithms process race-related data; and (4) establishing accountability measures that prioritize equitable outcomes for Black patients. By integrating these principles, AI can be developed to produce more equitable and culturally responsive healthcare interventions. This anti-racist approach challenges policymakers, researchers, clinicians, and AI developers to fundamentally rethink how AI is created, used, and regulated in healthcare, with profound implications for health policy, clinical practice, and patient outcomes across all medical domains.

## 1 Introduction

Artificial Intelligence (AI) and machine learning (ML) technologies are rapidly transforming psychiatric care, offering unprecedented opportunities for early diagnosis, personalized treatment, and improved patient outcomes (1, 2). However, as these technologies become increasingly integrated into mental health services, there is a growing concern that they may inadvertently perpetuate or even exacerbate existing racial disparities, particularly for Black Americans (3–6). This Perspective argues for the urgent need to develop and implement anti-racist AI in healthcare, using psychiatric care as a lens to examine how systemic and multigenerational American anti-Black racism affects mental health outcomes and healthcare delivery.

Recent studies have highlighted the potential for AI systems to exhibit racial bias, even when race is not explicitly included as a variable (7–9). This phenomenon, often referred to as “algorithmic bias,” encompasses a range of deviations from normative standards, including statistical inaccuracies and ethical concerns (10). Algorithmic bias can emerge from multiple sources, such as biased training data, inadequate representation of minority groups in AI development teams, and failure to account for the broader sociocultural context in which these technologies are deployed (5, 11). While some biases may be technical artifacts, others reflect deeply entrenched structural inequities that AI systems inadvertently reproduce.

In the field of psychiatry, where diagnosis and treatment often rely heavily on subjective assessments and cultural nuances, the risk of perpetuating racial biases through AI is particularly acute (6, 12). The poorer performance of current clinical algorithms perpetuates the misdiagnosis and underdiagnosis of mental health conditions in Black Americans, largely due to their failure to account for the pervasive impacts of racism on mental health. This algorithmic bias reflects longstanding clinical tendencies to underestimate the psychological toll of systemic racism, leading to inadequate detection and treatment of mental health issues in Black American populations (13, 14).

While social and cultural factors impact health outcomes across medical domains, psychiatry particularly grapples with diagnostic constructs that are highly influenced by these factors. The absence of clear biological markers for most psychiatric disorders complicates AI-driven diagnostic predictions, necessitating careful consideration of how racial disparities manifest uniquely in psychiatric diagnoses (15, 16). For instance, the reliance on subjective clinical assessments makes psychiatric AI models particularly vulnerable to encoding racialized diagnostic tendencies rather than actual disease pathology (17). While structural racism pervades all areas of medicine, psychiatry's classification challenges introduce an additional layer of complexity, as the boundaries between pathology, cultural expression, and systemic bias remain highly contested (17–19). This means that anti-racist AI governance in psychiatry cannot simply borrow strategies from other fields, it must actively account for the ways in which psychiatric diagnoses themselves are shaped by racialized assumptions and sociopolitical context (20).

One critical yet underexplored factor in these disparities is racial heteroscedasticity, which refers to the unequal variance in health outcomes and algorithmic predictions across racial groups, which may be driven by differential exposure to multigenerational American anti-Black racism-related stress (21). Because Black Americans are subject to systemic racism across multiple domains of life, their health-related experiences may demonstrate higher variability, leading to greater inconsistency in clinical algorithm performance (21). Moreover, beyond present-bound notions of racially-mediated stress, socially-mediated health detriments may accrue across generations, as the poor physical and mental wellbeing of parents has been shown to negatively impact the wellbeing of their children through possible mechanisms such as allostatic load and weathering (22–24). Failure to account for this variability reinforces disparities in psychiatric AI models.

Intersectionality and intergenerationality help explain this heteroscedasticity by identifying which minoritized individuals are more or less vulnerable to racism-related stress (21, 24, 25). Some subgroups, due to factors such as class privilege, skin tone, or generational status, may have stress exposures that resemble the lower variance patterns seen in majority group members, while others experience compounded disadvantages that amplify variability in health outcomes. Thus, intersectionality and intergenerationality provide a framework for understanding the social mechanisms behind racial heteroscedasticity, while heteroscedasticity itself describes the statistical consequences of these disparities in predictive modeling.

The impact of racism on mental health is well-documented in the research literature, with Black Americans experiencing disproportionately higher rates of psychological distress, chronic stress, and untreated mental illness compared to their white counterparts (26, 27). These disparities stem from a complex interplay of factors, including economic marginalization, limited access to care, cultural stigma, and the persistent effects of historical and ongoing racial discrimination (28, 29). This Perspective aims to address the challenges of modeling and eventually tackling these issues by proposing a framework for developing anti-racist AI in psychiatric care. There is a current wave of interest to remove race entirely from clinical algorithms (30, 31). We argue that effective AI systems in this field must go beyond mere “race-blind” approaches and actively work to counteract the effects of systemic racism on mental health outcomes (32, 33). In that light, it is important to note that contemporarily-identified Black Americans are a diverse racial subgroup comprised of people of African descent with vastly different generational identities within American society. The majority of today's Black Americans possess ancestry that originates within foundational American history, termed Ethnic Black Americans (EBAs) under the Sociohistorical Justice framework, who are the subgroup of Black Americans whose lineages carry the fullest intergenerational transmission of American anti-Black racism (34). In addition to EBAs, a growing number of today's Black Americans descend from lineages that voluntarily established within the US many centuries after American slavery and who do not possess familial ties to foundational American history. Despite sharing a present-day racial identity, the generationally-transmitted racism-related risk factors owing to American anti-Black racism are different between EBAs and other Black Americans. Therefore, as will be argued within this Perspective, our field must also go beyond “race-only” approaches to AI governance that homogenize centuries of differential exposure and generational transmission of health detriments born of American anti-Black racism. In suit, our proposed framework emphasizes the importance of incorporating measures of historical identity, cultural identity, resilience, and empowerment in AI models, recognizing these factors as crucial moderators of the relationship between racial stress and mental health outcomes (35, 36). We outline key principles for developing anti-racist psychiatric AI, including inclusive development processes, rigorous bias audits, and accountability measures that prioritize equitable outcomes for Black patients (37, 38).

By challenging researchers, clinicians, and AI developers to reimagine how we create and implement AI in psychiatry, this Perspective aims to contribute to a more equitable and historically informed approach to mental health care. In doing so, we hope to spark a broader conversation about the role of AI in addressing—rather than exacerbating—racial disparities in mental health and to pave the way for more just and effective psychiatric care for all.

## 2 Current limitations in AI models for Black patients

The rapid integration of AI and machine learning models into healthcare, particularly in psychiatry, has brought to light significant limitations in their performance for Black American patients. One of the seminal papers highlighting this issue was the 2019 study by Obermeyer et al., which revealed racial bias in a widely used healthcare algorithm (8). The study revealed that the algorithm rated Black patients as having the same risk level as White patients who were, in reality, in poorer health. This resulted in Black patients with complex health needs receiving reduced access to care (8). This work exposed how seemingly race-blind variables can perpetuate racial disparities in AI-driven healthcare decisions. While the Obermeyer study focused on a specific algorithm, it underscored a broader problem of racial bias in AI healthcare applications.

These biases may stem from various factors deeply rooted in generationally-compounded and presently-mediated systemic racism, as well as methodological shortcomings in AI development and deployment. One of the primary issues is the underrepresentation of Black individuals in the datasets used to train AI models. For example, in dermatology, a field where skin color plays a crucial role in diagnosis, studies have shown that out of 136 analyzed papers, only one explicitly included Black patients in their datasets, yielding worsened outcomes for Black patients in the AI detection of melanoma (39). This underrepresentation extends to psychiatry, where the lack of diverse representation in mental health research has significant implications for machine learning models' accuracy and equity. Studies show that psychiatric research often fails to adequately include Black participants, leading to unrepresentative datasets (40, 41). Black American youth are less likely to receive mental health treatment across various sources (42), resulting in skewed clinical datasets. The shortage of Black mental health professionals further compounds these issues, reducing culturally competent data collection and interpretation (43). Addressing these foundational issues of data representation is crucial for developing anti-racist AI in psychiatry. Beyond assuring race-based representation of Black American researchers and participants, due to the intergenerational impacts of American anti-Black racism, the Sociohistorical Justice framework equally calls attention for healthcare systems to attune to the differential historical identities of Black Americans, with specific attention called to ensuring commensurate representation of EBAs within healthcare practice (34).

In addition to data representation issues, recent research has uncovered more nuanced challenges in AI model performance for Black patients. Recent studies have demonstrated that even when machine learning models are trained exclusively on data from Black subjects, they still show lower predictive accuracy for this group compared to white subjects (6). This persistent disparity, even when controlling for socioeconomic factors, suggests that there are underlying mechanisms related to the experience of racism that are not being captured by current modeling approaches (6). We hypothesize that this phenomenon may be due to differences in variance across differential historical identities of contemporary racial groups. Specifically, we propose that EBA subgroups bear the synergistic effects of both historically-compounded and presently-mediated American anti-Black racism, which stands in contrast to other Black American subgroups whose lineages do not carry the historically-compounded elements of American anti-Black racism (34). Thus, Black Americans without generationally-transmitted American anti-Black trauma may exhibit variance more similar to that observed among the White patient group due to the absence of intergenerational detriments owing to anti-Black racism, and other historically-mediated stressors within a US social context. This increased variance may stem from varied exposure and psychological responses to historically-compounded racism-related stress among various generational identities of contemporary Black Americans (14, 27, 44). Importantly, racial heteroscedasticity violates fundamental assumptions of linear regression models (45–49), which are still widely used in both health disparities research and psychiatric prediction algorithms. Several high-impact papers have recently been published that demonstrate the failures of these linear models in generalizing to minoritized-population datasets (5, 6, 50); again, even those linear ML models trained solely on Black patient sample data (6). This reflects the need to further disaggregate historically-mediated risk factors of intergenerational American anti-Black racism.

**Figure 1:**
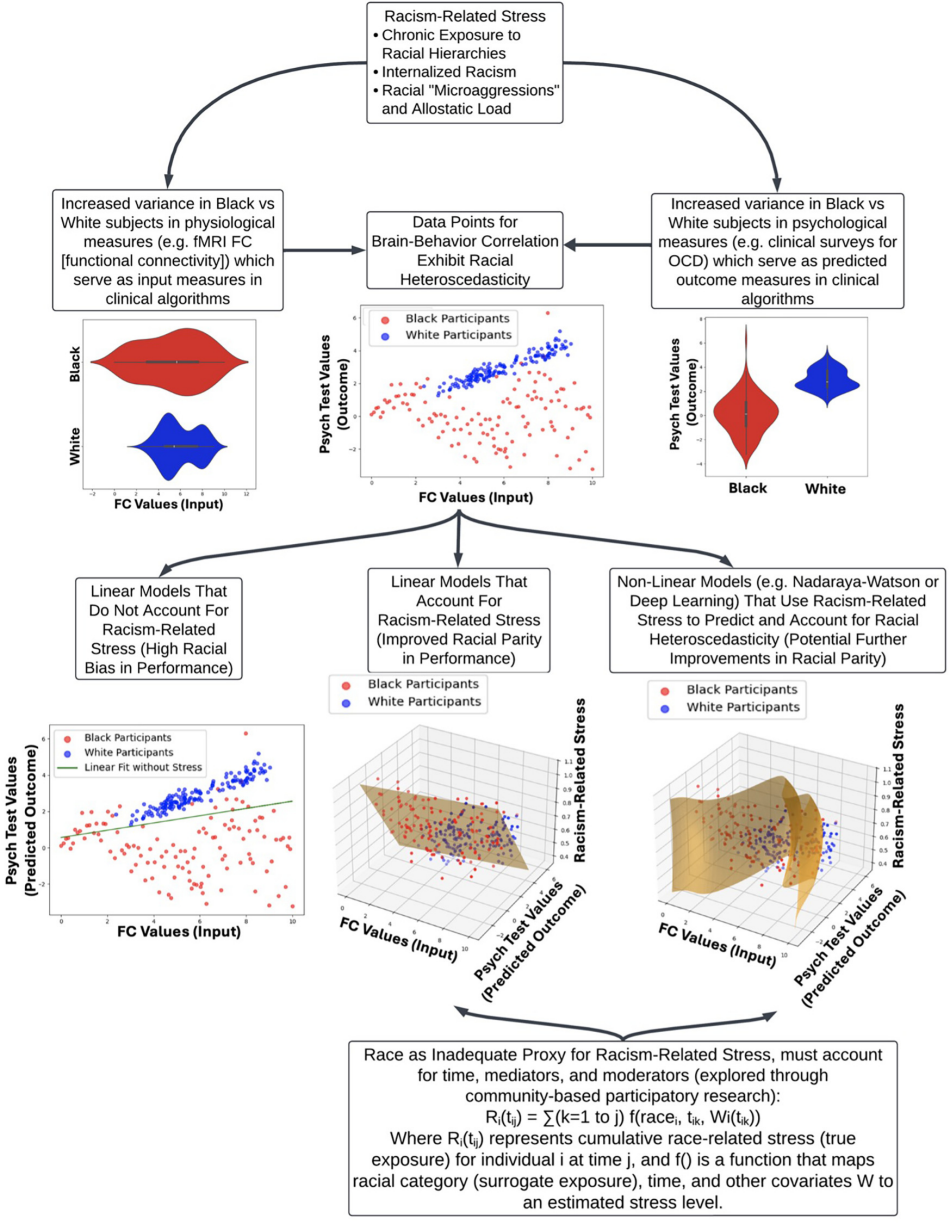
Impact of racism-related stress on clinical algorithm performance and racial heteroscedasticity. This flow chart illustrates how racism-related stress contributes to health disparities and affects clinical algorithm performance, focusing on the stress of racialization as a central factor. The top node introduces three key components of racism-related stress: chronic exposure to racial hierarchies, internalized racism, and the cumulative burden of racial microaggressions (allostatic load). Exposure to racism-related stress has wide-ranging impacts on targeted individuals, yielding a range of historically-compounded and non-historically-compounded subpopulations. This is reflected in the increased variance in physiological (e.g., fMRI functional connectivity) and psychological measures (e.g., clinical surveys for OCD) between Black and White subjects, which leads to racial heteroscedasticity in brain-behavior correlations. The lower section compares different modeling approaches: linear models without racism-related stress show significant racial bias, while incorporating stress into linear and non-linear models improves racial parity. The figure underscores the inadequacy of race as a sole proxy for racism-related stress, advocating for a more nuanced approach that includes time, mediators, and moderators, supported by community-based participatory research (CBPR).

Figure 1 provides a visual framework for how racial heteroscedasticity potentially impacts model accuracy across sociohistorical and racial groups. In the lower left panel, standard linear models fail to capture the complexity of variance in stress exposure, leading to biased predictions. This aligns with empirical findings demonstrating that traditional models tend to misclassify high-variability subgroups, particularly in datasets reflecting racialized health disparities. The lower middle and lower right panels shows how incorporating racism-related stress as a moderating factor in both linear and non-linear models improves parity in algorithmic outcomes. This suggests that AI models must explicitly account for historically-compounded and directly-experienced American anti-Black racism-related stress to mitigate racial disparities in predictive accuracy. Additionally, the observed heteroscedasticity may indicate the presence of “historically-compounded” and “non-historically-compounded” Black subpopulations, reinforcing the need for tailored modeling approaches that account for intra-group variation rather than treating racialized groups as sociohistorical monolithic entities.

Beyond linear models, non-linear AI architectures such as deep neural networks and ensemble models may also fail to account for racial heteroscedasticity if training data do not sufficiently capture the full range of stress-related variability (21). These models, which optimize performance across the entire population rather than subgroups, often smooth out or obscure race-related and intergenerational-related variance rather than explicitly modeling it. This failure is especially problematic in clinical AI, where the cumulative impact of racism-related stress produces distinct physiological and psychological signatures in different sociohistorical subpopulations within contemporarily racialized groups.

Additionally, there is a concerning trend of AI models amplifying existing biases in clinical practice. For example, if historical data reflect a pattern of underdiagnosis or misdiagnosis of certain mental health conditions in Black populations, AI models trained on these data may perpetuate and even exacerbate these errors (51). Several prior studies reported lower lifetime risk of psychiatric disorders for Black individuals compared to whites, a finding that may reflect underdiagnosis or cultural differences rather than true lower prevalence (52–55). This issue is particularly salient given the complex relationship between race, clinician bias, and mental health diagnoses. Barnes and Bates (2017) highlight a paradox where Black Americans often show lower diagnostic prevalence of major depressive disorder compared to White Americans in epidemiological studies, despite experiencing higher levels of psychological distress and exposure to risk factors (56). Moreover, Jegarl et al. (2023) highlighted mechanisms by which Black Americans may also have their depressive and substance use symptoms misdiagnosed as psychosis (57). These discrepancies suggest potential underdiagnosis or misdiagnosis of depression in Black populations, which may be moderated by clinician racialized bias and lead to downstream disparities in treatment services offered to Black patients (20). Furthermore, Black and Calhoun (2022) argue that biased and carceral responses to racially-minoritized persons with mental illness in acute medical care settings can constitute iatrogenic harms, potentially leading to further misdiagnosis and inadequate treatment (58). These biases, when incorporated into AI models, risk perpetuating and amplifying racial disparities in mental health care, underscoring the critical need for careful consideration of how race and mental health data are used in AI development.

There is currently a great deal of debate about the removal of race as a factor in clinical models (30, 31). The use of race as a simplistic binary variable in many AI models fails to capture the complex, multidimensional nature of racial identity and the differential historical identities within contemporarily racialized groups, and their synergized impact on health outcomes. This reductionist approach can perpetuate harmful stereotypes and lead to overgeneralized conclusions about Black patients' health needs and risks (33). Moving forward, it is critical that we shift away from using race as an insufficient proxy of history-based or present-day risk and instead focus on identifying and measuring the factors that mediate and modify racism-related stress. This will require a concerted effort to develop anti-racist AI approaches that can capture the nuanced impacts of structural racism, discrimination, and chronic stress on mental health outcomes that may be compounded through generational transmission in a given social context. By substituting more precise measures of intergenerational and race-related stress for crude racial categories, we may be able to build models that more accurately reflect the lived experiences of Black patients and avoid perpetuating harmful biases. Achieving this goal will take dedicated research to elucidate the complex pathways through which racism affects mental health over time and across generations, as well as collaboration between data scientists, clinicians, and communities to ensure new AI tools are equitable and patient-centered. While challenging, this paradigm shift is essential for developing psychiatric AI systems that can help reduce, rather than exacerbate, racial disparities in mental health care.

## 3 Directly-experienced, American anti-Black racism-related stress as a key contributor to mental health outcomes

The impact of directly-experienced, American anti-Black racism-related stress on mental health outcomes is a critical factor that must be accounted for in the development of anti-racist AI models in psychiatry. Racism-related stress refers to the cumulative psychological and physiological effects of experiencing chronic discrimination, microaggressions, and systemic oppression (27). This stress has been shown to have significant negative impacts on mental health outcomes for Black Americans, contributing to higher rates of depression, anxiety, and post-traumatic stress disorder (PTSD) (28), but has also been linked to a wide range of health outcomes including hypertension, cardiovascular disease, obesity, and accelerated cellular aging as measured by telomere length (59–61). The Everyday Discrimination Scale, a widely used measure of perceived discrimination, has been associated with numerous physical health outcomes, including coronary artery calcification, sleep disturbances, and chronic pain (62–64).

A crucial sociological insight into the importance of centering the impact of racism on mental health predictive models comes from the Anderson (2022) “Black in White Space” framework (12). This comprehensive ethnographic work, spanning over 45 years (12, 65–68), illuminates how structural racism operates in everyday life. Anderson's research underscores the psychological toll on Black Americans navigating predominantly white environments, where they must constantly be prepared for potential discrimination or hostility. The concept of “Black in White Space” emphasizes that the “Black ghetto” is not simply a physical space but has become an icon and a deep source of prejudice, negative stereotypes, and discrimination. Because of the power of this “iconic ghetto” and the lingering impact of systemic racism, Black people are typically burdened by a negative presumption they must disprove before establishing trusting relations with others. This challenge contributes to a wide range of racial disparities, including in healthcare, employment, education, police contact, incarceration, joblessness, housing, and random insults in public. Burdened with a deficit of credibility, Black people are often required to “dance,” or to perform respectability before a largely unsympathetic audience whose minds are typically already made up about where the Black person belongs — long before they belong in the white space, they are assumed to belong in the Black space. This process of negotiation through social interaction is a constant stressor that AI models must account for when assessing mental health risks and outcomes for Black Americans. The chronic stress and vigilance required in these contexts can contribute significantly to what has been termed “racial weathering,” with deleterious effects on mental health that current AI models often fail to capture.

The concept of “weathering,” introduced by Geronimus (1992), posits that the cumulative impact of racism and socioeconomic disadvantage leads to accelerated biological aging and increased allostatic load (69). This would manifest in higher rates of stress-related physical and mental health conditions among Black Americans compared to their white counterparts. Gee et al. (2019) emphasize the importance of time in understanding racism's effects on health (13), considering three key dimensions: time as age, exposure, and resource/privilege. Regarding time as age, Black Americans may experience accelerated aging due to chronic stress, evidenced by earlier onset of disease (70), greater morbidity at younger ages (71), shorter telomere lengths (59, 72), and shorter life expectancy compared to White individuals (73). Recent studies have directly linked experiences of racial discrimination to accelerated epigenetic aging and increased depressive symptoms, providing compelling evidence for the weathering hypothesis in the context of mental health outcomes (74). Regarding exposure, the duration, frequency, and timing of racism can significantly impact health outcomes, with potential critical periods during the life course. For instance, perceived racial discrimination during adolescence predicted depressive symptoms in young adulthood, even after controlling for earlier symptoms (75). Furthermore, time itself is a racialized resource, with racially minoritized people often experiencing a “time penalty” in various aspects of life. This inequitable distribution of time can exacerbate stress and contribute to poor health outcomes. Integrating these temporal dimensions provides a more nuanced understanding of how systemic racism affects mental health over the life course.

## 4 Historically-transmitted, anti-Black racism-related stress as a key contributor to mental health outcomes

The multigenerational impact of racism on mental health is not uniform across Black American sociohistorical subgroups. Factors such as generational exposure to American anti-Black racism, immigration status, and acculturation processes can influence an individual's risk owning to historically-transmitted racism-related stress (14). Shervin Assari's work on Marginalization-related Diminished Returns (MDR) further complicates this picture by demonstrating that socioeconomic status (SES) interacts with racial identity in surprising ways. Contrary to expectations, Assari and colleagues have found that higher SES often fails to protect Black Americans from poor health outcomes to the same degree it does for White Americans (76). For instance, education level has been shown to have a weaker protective effect against depression for Black adults compared to White adults (77). Similarly, income has been found to have a weaker association with self-rated health for Black individuals than for White individuals (78). These findings suggest that racism not only directly impacts health but also reduces the protective effects of socioeconomic resources. Furthermore, research has shown that the health effects of discrimination can vary based on historical subgroup. Namely, Black Americans (being Ethnic Black Americans according to the sociohistorical justice framework) (34) and U.S.-born Black American individuals, being Black Americans who grew up in America's white dominant society, may report more discrimination and associated health impacts than Caribbean Black individuals or recent African immigrants, who are Black Americans who grew up in predominantly Black societies and/or societies where race is not a dominant sociopolitical construct (79, 80). Additionally, factors such as racial identity strength, coping strategies, and social support have been found to moderate the relationship between perceived discrimination and mental health outcomes (81–83).

In light of the complex and nuanced ways in which racism impacts health outcomes for historically-diverse Black Americans, it is critical that AI healthcare models incorporate a sophisticated understanding of racism-related stress. Furthermore, to truly account for the historically-transmitted vs. directly-experienced nuances of racism, AI healthcare models must be individually tailored to each society's unique historical, sociopolitical construction of race. That is, the intergenerational health implications of anti-Black racism cannot be meaningfully generalized from an American historical context to a different white dominant social context, like Canada or England, because those countries do not have a foundational population of Black Canadians or Britons since enslavement (84–86). Similarly, intergenerational health implications cannot be automatically generalized to Black-normative social contexts where anti-Blackness is not a dominant sociopolitical construct to determine social privilege, like the Caribbean or West Africa (87, 88). The evidence presented demonstrates that racism affects health through multiple pathways, including direct physiological impacts of chronic stress, reduced returns on protective factors like education and income, and varied effects across different sociohistorical subgroups of Black Americans. Simply including race as a variable in AI models is insufficient and may even perpetuate harmful biases. Instead, developers of AI healthcare models must strive to incorporate measures of racism-related stress, consider the moderating effects of factors like racial identity and coping strategies, and account for the differential impacts of socioeconomic status across racial groups. By doing so, these models can more accurately reflect the historically-diverse lived experiences of Black Americans and provide more equitable and effective healthcare recommendations. This nuanced approach is essential for improving racial parity in AI healthcare models and, ultimately, for addressing the persistent health disparities that affect Black communities in the United States.

## 5 Key principles for developing anti-racist psychiatric models

The development of anti-racist psychiatric AI models requires a fundamental shift in approach, prioritizing the inclusion of diverse perspectives throughout the entire process. A critical aspect of this shift is the involvement of community stakeholders in the development of racism-related stress measures that are incorporated into AI models. This inclusion is particularly crucial given the intersectional complexity and nuances of the impacts of racism on health outcomes, as discussed in the previous section. The varied experiences of racism across different Black sociohistorical subgroups, the moderating effects of factors such as socioeconomic status and immigration status, and the temporal dynamics of racism-related stress all contribute to a complex landscape that cannot be adequately captured without direct input from affected communities. As illustrated in Figure 1, the mathematical modeling of historically-compounded race-related stress must account for multiple factors, including time dimensions, intersectional moderating identities, and the use of race as a proxy for directly experienced racism.

**Figure 2:**
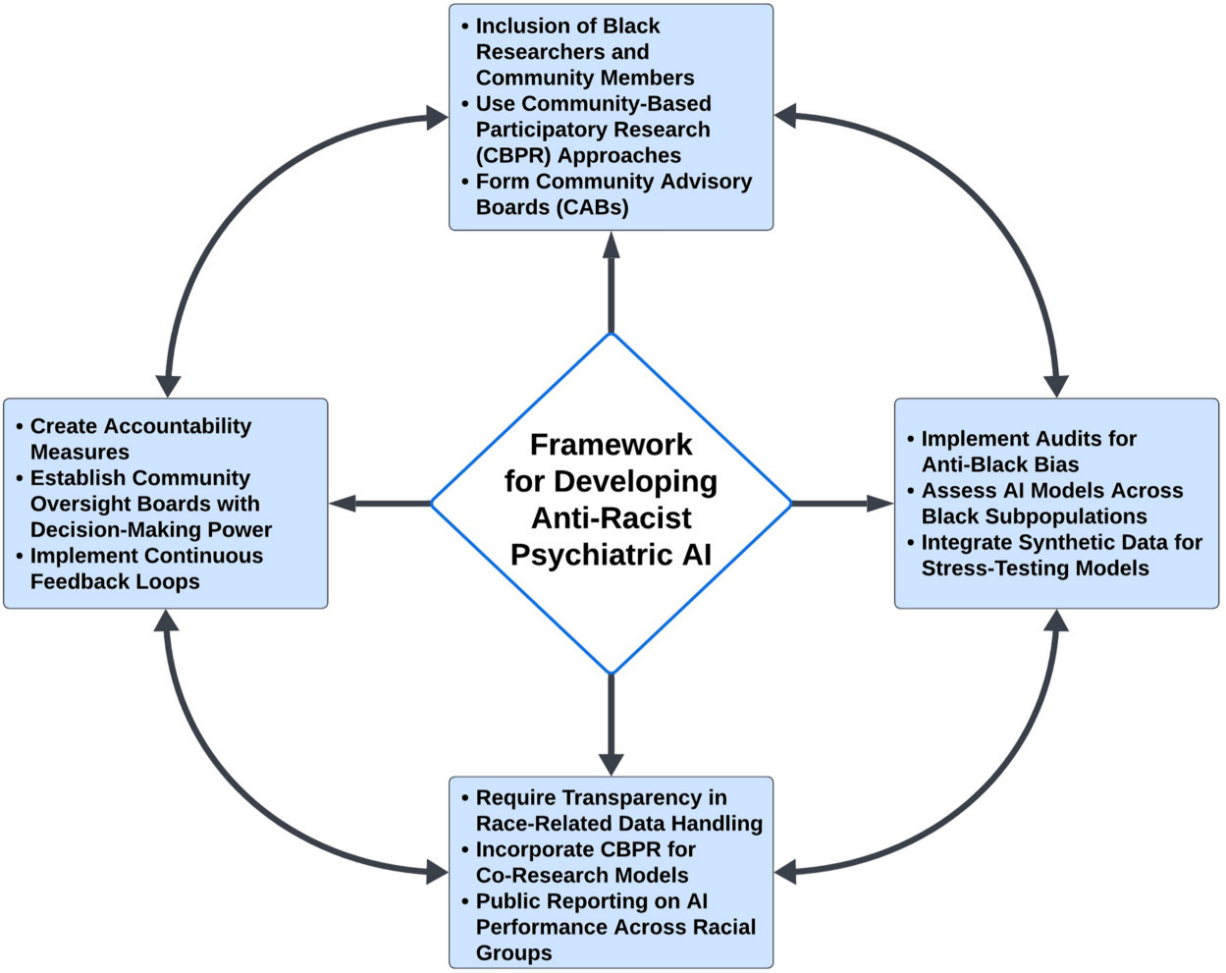
Framework for Developing Anti-Racist Psychiatric AI. The framework for developing anti-racist psychiatric AI models emphasizes the inclusion of diverse perspectives and experiences throughout the entire process. It begins with a mandate to include Black researchers and community members, ensuring that AI models accurately reflect the lived experiences and priorities of those they serve. Community-based participatory research (CBPR) plays a central role, facilitating the creation of culturally relevant and responsive AI models. The framework also underscores the importance of rigorous audits to assess for anti-Black bias, transparency in the handling of race-related data by algorithms, and the establishment of robust accountability measures. These components work together to ensure that psychiatric AI systems contribute to equitable mental health outcomes for Black patients, transforming AI from a potential source of bias into a tool for justice and equity in mental healthcare.

By involving community members in the development of these measures, we can ensure that the AI models more accurately reflect the lived experiences of diverse Black Americans and capture the nuanced ways in which racism impacts mental health. This collaborative approach not only improves the validity and reliability of the measures, as has been observed for other patient-centric research (89), but also helps to build trust between researchers and communities, which is essential for the successful implementation and adoption of AI technologies in mental health care. In addition, the following key principles should be incorporated toward the development of anti-racist AI, as illustrated in Figure 2.

### 5.1 Mandate inclusion of Black researchers and community members in AI development

The development of anti-racist psychiatric AI models necessitates the active inclusion of Black researchers and community members throughout the entire process (37). This principle goes beyond mere tokenism and calls for a fundamental shift towards community-based participatory research (CBPR) approaches in AI development (90). By including researchers with sociohistorically-diverse lived experience of anti-Black racism, along with engaging Black communities as co-researchers, we can ensure that the AI models reflect the lived experiences and priorities of those they aim to serve. CBPR approaches have shown significant promise in addressing health disparities and developing culturally relevant interventions (37, 91). In the context of psychiatric AI, this could involve forming community advisory boards (CABs) comprising diverse Black community members, mental health professionals, and researchers to guide the development process. These CABs can provide invaluable insights into the nuanced ways racism impacts mental health, help identify culturally specific protective factors, and ensure that the AI models are responsive to community needs and values (37).

Moreover, it is crucial to recognize and explore psychological protective factors that may vary across different Black American sociohistorical subgroups (92, 93). This exploration necessitates the involvement of sociohistorically-diverse Black researchers and community members who can provide invaluable insights into these nuanced experiences. For instance, research has shown that first-generation Black immigrants from Black-normative societies often demonstrate better mental health outcomes compared to their U.S.-born counterparts (79). This “immigrant paradox” may be attributed to stronger cultural identity, community connectedness, and different experiences with racism (94, 95). However, these protective effects tend to erode over time and generations as immigrants acculturate to being “Black in White Space” (12). The involvement of diverse historical subgroups within the Black American community is necessary to capture these nuances. AI models must be designed to account for these temporal dynamics and the varying impacts of racism across generations, which can only be achieved through the active participation of sociohistorically-representative Black researchers and community members who have lived these experiences.

The IMANI Breakthrough project provides an instructive example of how CBPR principles can transform healthcare interventions for marginalized communities (96). This substance use intervention for Black and Latinx communities began with nine months of relationship-building through community meetings to develop research questions and implementation strategies. Through learning conversations in churches across Connecticut, researchers discovered how structural racism and discriminatory policies shaped community needs around treatment. This deep community engagement led to substantial outcomes: a 42% retention rate compared to reported outpatient treatment completion rates of 20.4% for Black individuals and 14.7% for Latinx individuals in traditional programs (97). IMANI researchers demonstrated, working directly with community members, how standardized health measures needed to be adapted to capture community-specific experiences of social determinants of health (96).

Similar principles should guide AI development in psychiatry. Just as IMANI spent months building community relationships before intervention design, AI developers should engage with affected communities well before algorithm development begins. Like IMANI's community-driven adaptation of wellness measures, AI researchers need to work with communities to develop more culturally responsive ways of measuring and modeling mental health outcomes that capture the impacts of racism-related stress. While this approach requires greater upfront investment in relationship-building, the IMANI project demonstrates how such engagement ultimately produces more effective and equitable interventions (96).

### 5.2 Implement audits that specifically assess for anti-Black bias

To ensure that psychiatric AI models do not perpetuate racial disparities, it is crucial to implement rigorous and ongoing audits specifically assessing for anti-Black bias that is sociohistorically-nuanced. These audits should transcend simple measures of overall model performance and probe the nuanced ways AI systems may discriminate against Black patients. Regular assessments of model performance and impact across different racial groups, particularly within various Black subpopulations, are essential. This includes evaluating the model's accuracy, fairness, and potential for harm across intersections of race, ethnicity, gender, age, and socioeconomic status within Black communities. Such comprehensive audits can uncover disparities in model performance that might be masked by aggregated data, which is a common issue when broad metrics are used without considering subgroup variabilities (26, 98–100).

The potential for AI models to amplify or reinforce existing biases in clinical practice should be another key focus of these audits. For example, these audits should determine whether model recommendations align with known patterns of under-diagnosis or misdiagnosis of mental health conditions in Black American populations. This involves evaluating how models handle culturally specific expressions of mental distress that may not conform to traditional diagnostic criteria (56).

Finally, it is imperative that audits for American anti-Black bias in psychiatric AI models may be both retrospective and predictive. This means that the audits should actively seek to forecast and preempt potential biases before they manifest in real-world clinical settings. One effective approach could be the integration of synthetic data representing under-represented Black subpopulations into the training and validation processes of these models (101, 102). By simulating scenarios where the AI might encounter diverse Black patients with complex intersecting identities (e.g., a young Ethnic Black American woman with a low socioeconomic status suffering from culturally specific expressions of distress), the audit can stress-test the model's fairness and accuracy across various contexts. This proactive strategy ensures that models are equipped to handle the rich diversity within Black American communities and reduces the risk of perpetuating existing biases, ultimately fostering more equitable mental health outcomes.

### 5.3 Require transparency in how algorithms handle race-related data

The lack of transparency in many AI algorithms, often protected as proprietary information, makes it difficult to identify and address potential biases. This “black box” nature of AI systems can obscure discriminatory practices and hinder efforts to improve model performance for Black American patients (103). To counteract this, it is essential to mandate transparency throughout the entire AI development lifecycle, particularly concerning the treatment of race-related data. Given the historical context, where Black American communities have been exploited and misled in research, such as during the Tuskegee Syphilis Study (104), it is imperative that the development and employment of the most explainable models be prioritized for use in clinical settings (105). This step is vital in fostering trust, especially within Black communities that have historically been marginalized in healthcare systems, such as Ethnic Black American communities.

Central to achieving this transparency is the incorporation of CBPR principles and co-research models in AI development. CBPR emphasizes the involvement of community members as equal partners in the research process, from the initial stages of algorithm design to the final deployment in clinical settings (37). By engaging Black American communities directly in the development and evaluation of AI models, researchers can ensure that the models are not only explainable but also culturally relevant and aligned with the needs and concerns of those they are designed to serve. This collaborative approach not only enhances the transparency and explainability of AI but also empowers Black American communities, giving them agency in the tools that affect their healthcare. Furthermore, co-research fosters a deeper understanding of the social determinants that influence health disparities, allowing AI models to account for these factors more effectively (37, 106). This engagement is crucial for transforming AI from a potential source of bias into a tool for equity and justice in mental healthcare.

Finally, routine public reporting on the performance of AI systems across different racial and historical groups is essential for building trust and accountability. These reports should be accessible to the general public and presented in a format that is easy for non-experts to understand. Engaging the community in discussions about AI transparency and equity, grounded in CBPR principles, will further strengthen the relationship between healthcare providers and Black American communities. This approach ensures that AI tools are used ethically and effectively to improve mental health outcomes for all patients, particularly those from historically marginalized groups.

### 5.4 Create accountability measures that prioritize equitable outcomes for Black American patients

While transparency is crucial, it must be coupled with robust accountability measures to ensure that psychiatric AI models actively work towards achieving equitable outcomes for Black American patients. This involves moving beyond mere oversight to implementing concrete mechanisms that hold developers, healthcare providers, and institutions responsible for the performance and impact of AI systems on Black mental health.

In addition to using CBPR principles in the development of clinical models, another critical accountability measure is the establishment of community oversight boards with decision-making power. These boards, comprised primarily of Black American community members and mental health professionals, would have the authority to approve or reject the use of AI models based on their potential impact on Black American mental health outcomes. This approach ensures that the community most affected by these technologies has a direct say in their implementation (107).

Moreover, to deepen accountability, it is essential to implement a system of continuous feedback loops between the AI developers and the communities they serve. This could involve the use of real-time data sharing where communities are regularly updated on the performance of AI models, and their feedback is actively sought and incorporated into ongoing model adjustments. Such a system not only places community voices at the center of AI development but also ensures that the models evolve in response to the lived experiences and needs of Black American patients. By making these feedback loops a mandatory aspect of AI model deployment, we can guarantee that these systems remain responsive and accountable to those they are designed to serve.

## 6 Conclusion

The development of anti-racist AI in psychiatry would represent a pivotal advancement for mental health care and serve as a model for addressing racial disparities across all healthcare domains. This approach has the potential to enable more accurate diagnoses and timely interventions tailored to the unique experiences of Black American patients and other marginalized groups throughout the healthcare system.

However, realizing the full potential of anti-racist AI will require overcoming significant challenges and enacting major culture shifts among American healthcare research. Key among these is the need for inclusive development teams, comprehensive data that captures the historically nuanced experiences of racism, and the application of advanced statistical methods that address racial heteroscedasticity. Furthermore, ongoing community engagement, rigorous ethical standards, and robust accountability measures must be central to the development process. By prioritizing these elements, we can ensure that AI not only serves as a tool for reducing disparities but also contributes to the broader goal of dismantling systemic inequities in mental health care.

While this paper focuses on psychiatric AI, its challenges and governance principles apply across medicine. In psychiatric AI, bias stems not just from underrepresentation in training datasets but also from failing to account for intergenerational racism-related stress as a determinant of brain function and behavior. This issue extends beyond psychiatry to numerous clinical algorithms that have been widely criticized for their poor performance in Black American patients, regardless of whether they currently use “race-correction” factors. The eGFR (Estimated Glomerular Filtration Rate), which includes a race-based correction factor, misestimates kidney function in Black patients, leading to delayed referrals for dialysis and transplantation (33, 108, 109). However, removing the race factor does not fully eliminate the racial disparities in CKD classification, as race-blind equations still result in lower eGFR estimates for Black American patients, increasing their likelihood of being diagnosed with CKD and reclassified into more severe disease stages compared to White patients (109). The Pooled Cohort Equations (PCE), used for cardiovascular risk assessment, differentially overestimates or underestimates risk based on race, contributing to inequitable allocation of preventive treatments (110, 111). Maternal health risk models consistently underestimate risks for Black American women, including for conditions like preeclampsia and postpartum hemorrhage, resulting in preventable maternal health disparities (31, 112, 113). Similarly, cancer risk prediction models, such as those for breast cancer, demonstrate lower accuracy in Black American women, exacerbating disparities in screening and early detection (114).

Beyond dataset underrepresentation, current clinical AI models fail to account for the broad health consequences of racism and racial socialization. Future clinical AI models must move beyond simplistic race adjustments and instead develop robust racism indices that leverage existing bodies of work that explicitly capture the historically-compounded biological and psychological consequences of American anti-Black racism. These include extensive research showing profound health correlates of David Williams' Everyday Discrimination Scale, Arline Geronimus' Weathering Hypothesis, Nancy Krieger's Ecosocial Theory, and Camara Jones' framework on levels of racism (69, 115–118). Relevant to a wide range of clinical models, studies have demonstrated that racial stress is embodied physiologically, accelerating allostatic load, neurobiological aging, and disease risk, particularly in Black Americans; however, these findings have yet to be fully integrated into clinical models (72, 119–121). These indices should complement existing social determinants of health (SDOH) measures, such as the Area Deprivation Index (ADI), and be further developed to account for sociohistorical context, ensuring that structural racism is formally integrated into risk assessment rather than erased as solely the effects of socioeconomic status (76, 122–124).

While clinical models fail to account for the impacts of American anti-Black racism within the context of US healthcare, similar biases may be present in other underrepresented groups, including Latinx populations and non-US contexts. Future work should explore how the concepts discussed here generalize across different healthcare systems and populations. In particular, the mechanisms underlying racial heteroscedasticity may vary depending on historical, economic, and policy-driven factors shaping racialization in different nations. For example, while US-based studies highlight the cumulative impacts of American anti-Black racism on psychiatric diagnosis and treatment, similar disparities exist in European or Latin American contexts through distinct but functionally equivalent pathways of medical neglect and epistemic injustice (18, 125–127). Caution must be exercised against generalization of AI healthcare models that have been calibrated for one sociopolitical and historical context to another. Nevertheless, expanding this work to international settings would help clarify how the AI governance principles outlined here can be optimally adapted for different sociopolitical and national landscapes.

## Data Availability

The original contributions presented in the study are included in the article, further inquiries can be directed to the corresponding author.
